# Tetra­aqua­bis­(tetra­zolido-κ*N* ^1^)magnesium

**DOI:** 10.1107/S1600536810027625

**Published:** 2010-07-21

**Authors:** Ti-Lou Liu, Ji-Hua Deng, Shuang-Jiao Sun

**Affiliations:** a Shaoyang Medical College, Shaoyang, Hunan 422000, People’s Republic of China; b College of Chemistry and Bio-engineering, Yichun University, Yichun, Jiangxi 336000, People’s Republic of China

## Abstract

In the crystal structure of the title compound, [Mg(CHN_4_)_2_(H_2_O)_4_], the Mg^II^ atom is six-coordinated by two N atoms from two tetra­zolide anions and four O atoms from four coordinated water mol­ecules in a slightly distorted octa­hedral geometry. The Mg atom is located on centres of inversion whereas the tetra­zolide anion and the water mol­ecules occupy general positions. The crystal packing is stabilized by intermolecular O—H⋯N hydrogen bonding between the tetra­zolide anions and the coordinated water mol­ecules.

## Related literature

For metal complexes with tetra­zolide anions, see: Zhang *et al.* (2007 ▶); He *et al.* (2006 ▶).
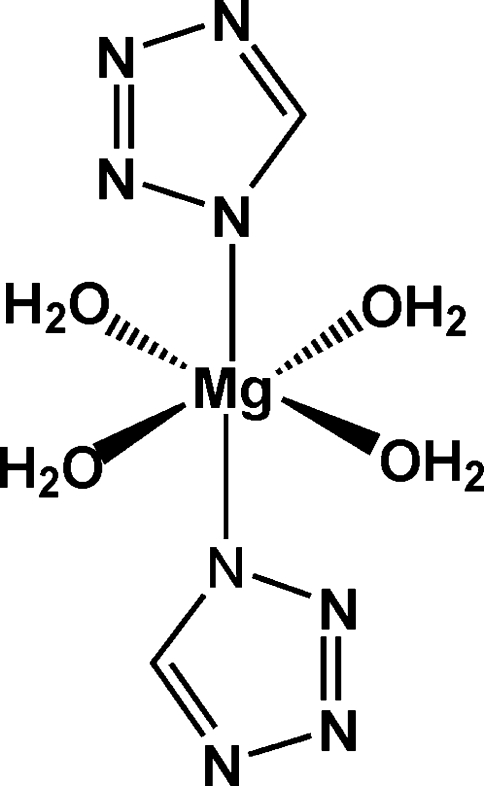


## Experimental

### 

#### Crystal data

[Mg(CHN_4_)_2_(H_2_O)_4_]
*M* *_r_* = 234.49
Monoclinic, 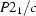

*a* = 5.7570 (19) Å
*b* = 11.638 (4) Å
*c* = 6.963 (2) Å
β = 99.785 (5)°
*V* = 459.7 (3) Å^3^
*Z* = 2
Mo *K*α radiation
μ = 0.21 mm^−1^
*T* = 173 K
0.36 × 0.28 × 0.22 mm

#### Data collection

Bruker SMART CCD area-detector diffractometer
Absorption correction: multi-scan (*SADABS*; Bruker, 1998 ▶) *T*_min_ = 0.929, *T*_max_ = 0.955
1806 measured reflections
792 independent reflections
709 reflections with *I* > 2σ(*I*)
*R*_int_ = 0.015

#### Refinement

*R*[*F* ^2^ > 2σ(*F* ^2^)] = 0.033
*wR*(*F* ^2^) = 0.097
*S* = 1.01
792 reflections
86 parameters
6 restraints
H atoms treated by a mixture of independent and constrained refinement
Δρ_max_ = 0.35 e Å^−3^
Δρ_min_ = −0.25 e Å^−3^

### 

Data collection: *SMART* (Bruker, 1998 ▶); cell refinement: *SAINT* (Bruker, 1998 ▶); data reduction: *SAINT*; program(s) used to solve structure: *SHELXS97* (Sheldrick, 2008 ▶); program(s) used to refine structure: *SHELXL97* (Sheldrick, 2008 ▶); molecular graphics: *SHELXTL* (Sheldrick, 2008 ▶); software used to prepare material for publication: *SHELXTL*.

## Supplementary Material

Crystal structure: contains datablocks I, global. DOI: 10.1107/S1600536810027625/nc2193sup1.cif

Structure factors: contains datablocks I. DOI: 10.1107/S1600536810027625/nc2193Isup2.hkl

Additional supplementary materials:  crystallographic information; 3D view; checkCIF report

## Figures and Tables

**Table 1:** Hydrogen-bond geometry (Å, °)

*D*—H⋯*A*	*D*—H	H⋯*A*	*D*⋯*A*	*D*—H⋯*A*
O2—H2*B*⋯N3^i^	0.83 (2)	1.96 (2)	2.7797 (19)	173 (2)
O1—H1*B*⋯N1^ii^	0.88 (2)	1.89 (2)	2.755 (2)	169 (2)
O1—H1*A*⋯N4^iii^	0.81 (2)	2.15 (2)	2.956 (2)	173 (2)
O2—H2*A*⋯N4^iv^	0.83 (2)	2.06 (2)	2.892 (2)	171 (2)

Symmetry codes: (i) 
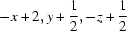
; (ii) 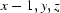; (iii) 
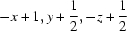
; (iv) 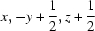.

**Fig. 1.**
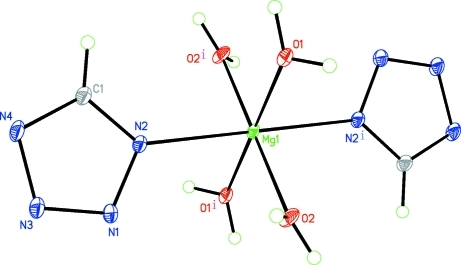
The structure of (I), showing 30% probability displacement ellipsoids and the atom-labeling scheme. Symmetry code: i = x,y,z.

**Fig. 2.**
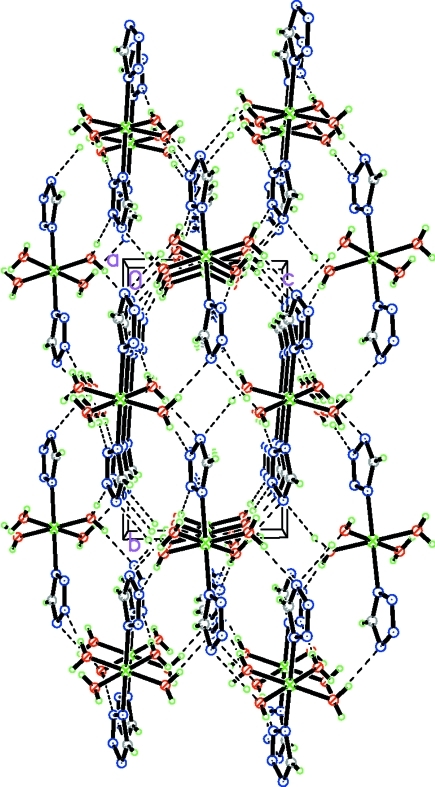
Crystal structure of title compound with view along the a axis. Hydrogen bonding is shown as dashed lines.

## supplementary crystallographic information

### Comment

Tetrazolide anions are found in a number of metal complexes as ligands and the crystal structures and properties of several of such metal complexes have been reported in literature (Zhang *et al.*, 2007; He *et al.*, 2006) In the present contribution we report the synthesis and crystal structure of it's magnesium(II) complex. In the crystal structure of the title compound the Mg atoms are six-coordinated by two N atoms from two symmetry equivalent tetrazolide anions and four O atoms of two pairs of symmetry equivalent water molecules. The coordination polyhedra around the Mg atoms can be described as slightly distorted tetrahedra (Fig. 1). In the crystal structure the complexes are connected by intermolecular O—H···N hydrogen bonding into a three-dimensional network (Fig. 2 and Tab. 1).

### Experimental

A solution of MgCl_2_ 2H_2_O (1 mmol) in water (5 ml) was slowly added to a solution of tetrazole (1 mmol) in water (14 ml) with continuous stirring at room temperature. After 30 minutes, the mixture was sealed in a 25 ml Teflon-lined stainless steel vessel and heated under autogenous pressure at 160 °C for 4 days, then slowly cooled to room temperature. The colorless crystals were collected by filtration, washed with distilled water and dried in air. Yield: 60% (based on Mg).

### Refinement

The H atoms of the tetrazole ligands were placed in geometrically idealized positions with C—H distances of 0.95 Å and were refined isotropic using a riding model with *U*_iso_(H) = 1.2Ueq(C). The H atoms of the coordinated water molecules were located in the difference Fourier maps and refined isotropic with varying coordinates.

### Crystal data

**Table:**

[Mg(CHN_4_)_2_(H_2_O)_4_]	*F*(000) = 244
*M**_r_* = 234.49	*D*_x_ = 1.694 Mg m^−^^3^
Monoclinic, *P*2_1_/*c*	Mo *K*α radiation, λ = 0.71073 Å
Hall symbol: -P 2ybc	Cell parameters from 1468 reflections
*a* = 5.7570 (19) Å	θ = 3.5–26.9°
*b* = 11.638 (4) Å	µ = 0.21 mm^−^^1^
*c* = 6.963 (2) Å	*T* = 173 K
β = 99.785 (5)°	Block, colorless
*V* = 459.7 (3) Å^3^	0.36 × 0.28 × 0.22 mm
*Z* = 2	

### Data collection

**Table:**

Bruker SMART CCD area-detector diffractometer	792 independent reflections
Radiation source: fine-focus sealed tube	709 reflections with *I* > 2σ(*I*)
graphite	*R*_int_ = 0.015
phi and ω scans	θ_max_ = 25.0°, θ_min_ = 3.5°
Absorption correction: multi-scan (*SADABS*; Bruker, 1998)	*h* = −5→6
*T*_min_ = 0.929, *T*_max_ = 0.955	*k* = −13→12
1806 measured reflections	*l* = −7→8

### Refinement

**Table:**

Refinement on *F*^2^	Primary atom site location: structure-invariant direct methods
Least-squares matrix: full	Secondary atom site location: difference Fourier map
*R*[*F*^2^ > 2σ(*F*^2^)] = 0.033	Hydrogen site location: inferred from neighbouring sites
*wR*(*F*^2^) = 0.097	H atoms treated by a mixture of independent and constrained refinement
*S* = 1.01	*w* = 1/[σ^2^(*F*_o_^2^) + (0.0716*P*)^2^ + 0.1838*P*] where *P* = (*F*_o_^2^ + 2*F*_c_^2^)/3
792 reflections	(Δ/σ)_max_ < 0.001
86 parameters	Δρ_max_ = 0.35 e Å^−^^3^
6 restraints	Δρ_min_ = −0.25 e Å^−^^3^

### Special details

**Table:**

Geometry. All e.s.d.'s (except the e.s.d. in the dihedral angle between two l.s. planes) are estimated using the full covariance matrix. The cell e.s.d.'s are taken into account individually in the estimation of e.s.d.'s in distances, angles and torsion angles; correlations between e.s.d.'s in cell parameters are only used when they are defined by crystal symmetry. An approximate (isotropic) treatment of cell e.s.d.'s is used for estimating e.s.d.'s involving l.s. planes.
Refinement. Refinement of *F*^2^ against ALL reflections. The weighted *R*-factor *wR* and goodness of fit *S* are based on *F*^2^, conventional *R*-factors *R* are based on *F*, with *F* set to zero for negative *F*^2^. The threshold expression of *F*^2^ > σ(*F*^2^) is used only for calculating *R*-factors(gt) *etc*. and is not relevant to the choice of reflections for refinement. *R*-factors based on *F*^2^ are statistically about twice as large as those based on *F*, and *R*- factors based on ALL data will be even larger.

### Fractional atomic coordinates and isotropic or equivalent isotropic displacement parameters (Å^2^)

**Table:**

	*x*	*y*	*z*	*U*_iso_*/*U*_eq_	
Mg1	0.5000	0.5000	0.0000	0.0139 (3)	
N1	0.8863 (2)	0.31027 (12)	0.0878 (2)	0.0188 (4)	
N2	0.6554 (2)	0.32630 (12)	0.02230 (19)	0.0154 (4)	
C1	0.5683 (3)	0.22168 (15)	−0.0099 (2)	0.0184 (4)	
H1	0.4070	0.2058	−0.0575	0.022*	
N4	0.7331 (2)	0.14132 (12)	0.0322 (2)	0.0199 (4)	
O1	0.2683 (2)	0.45460 (11)	0.18011 (19)	0.0187 (3)	
O2	0.7258 (2)	0.54196 (11)	0.25064 (18)	0.0196 (4)	
N3	0.9328 (3)	0.20068 (12)	0.0938 (2)	0.0200 (4)	
H2A	0.729 (4)	0.4945 (18)	0.341 (3)	0.033 (6)*	
H1A	0.256 (4)	0.5042 (18)	0.260 (3)	0.037 (7)*	
H1B	0.136 (4)	0.417 (2)	0.150 (3)	0.043 (7)*	
H2B	0.819 (4)	0.594 (2)	0.293 (4)	0.045 (7)*	

### Atomic displacement parameters (Å^2^)

**Table:**

	*U*^11^	*U*^22^	*U*^33^	*U*^12^	*U*^13^	*U*^23^
Mg1	0.0152 (4)	0.0058 (5)	0.0200 (5)	−0.0001 (3)	0.0007 (3)	−0.0002 (3)
N1	0.0187 (8)	0.0104 (8)	0.0262 (8)	0.0028 (6)	0.0011 (6)	0.0006 (6)
N2	0.0170 (7)	0.0095 (8)	0.0194 (8)	0.0003 (6)	0.0017 (6)	−0.0003 (5)
C1	0.0192 (8)	0.0115 (9)	0.0235 (9)	−0.0005 (7)	0.0006 (7)	−0.0002 (7)
N4	0.0242 (8)	0.0090 (8)	0.0255 (9)	0.0007 (6)	0.0015 (6)	−0.0007 (6)
O1	0.0201 (7)	0.0106 (7)	0.0260 (7)	−0.0027 (5)	0.0053 (5)	−0.0036 (5)
O2	0.0237 (7)	0.0105 (7)	0.0221 (7)	−0.0051 (5)	−0.0034 (5)	0.0019 (5)
N3	0.0227 (8)	0.0108 (7)	0.0256 (8)	0.0026 (6)	0.0014 (6)	−0.0002 (6)

### Geometric parameters (Å, °)

**Table:**

Mg1—O1^i^	2.0492 (13)	N2—C1	1.321 (2)
Mg1—O1	2.0492 (13)	C1—N4	1.329 (2)
Mg1—O2^i^	2.0499 (13)	C1—H1	0.9500
Mg1—O2	2.0499 (13)	N4—N3	1.347 (2)
Mg1—N2	2.2053 (15)	O1—H1A	0.812 (19)
Mg1—N2^i^	2.2053 (15)	O1—H1B	0.876 (19)
N1—N3	1.302 (2)	O2—H2A	0.83 (2)
N1—N2	1.343 (2)	O2—H2B	0.83 (2)
			
O1^i^—Mg1—O1	180.00 (7)	N3—N1—N2	109.45 (13)
O1^i^—Mg1—O2^i^	85.69 (6)	C1—N2—N1	104.74 (13)
O1—Mg1—O2^i^	94.31 (6)	C1—N2—Mg1	134.05 (11)
O1^i^—Mg1—O2	94.31 (6)	N1—N2—Mg1	121.16 (10)
O1—Mg1—O2	85.69 (6)	N2—C1—N4	112.04 (15)
O2^i^—Mg1—O2	180.00 (7)	N2—C1—H1	124.0
O1^i^—Mg1—N2	88.91 (5)	N4—C1—H1	124.0
O1—Mg1—N2	91.09 (5)	C1—N4—N3	104.34 (15)
O2^i^—Mg1—N2	91.86 (5)	Mg1—O1—H1A	112.3 (16)
O2—Mg1—N2	88.14 (5)	Mg1—O1—H1B	128.1 (16)
O1^i^—Mg1—N2^i^	91.09 (5)	H1A—O1—H1B	110 (2)
O1—Mg1—N2^i^	88.91 (5)	Mg1—O2—H2A	114.5 (16)
O2^i^—Mg1—N2^i^	88.14 (5)	Mg1—O2—H2B	139.2 (17)
O2—Mg1—N2^i^	91.86 (5)	H2A—O2—H2B	106 (2)
N2—Mg1—N2^i^	180.0	N1—N3—N4	109.42 (14)

Symmetry codes: (i) −*x*+1, −*y*+1, −*z*.

### Hydrogen-bond geometry (Å, °)

**Table:**

*D*—H···*A*	*D*—H	H···*A*	*D*···*A*	*D*—H···*A*
O2—H2B···N3^ii^	0.83 (2)	1.96 (2)	2.7797 (19)	173 (2)
O1—H1B···N1^iii^	0.88 (2)	1.89 (2)	2.755 (2)	169 (2)
O1—H1A···N4^iv^	0.81 (2)	2.15 (2)	2.956 (2)	173 (2)
O2—H2A···N4^v^	0.83 (2)	2.06 (2)	2.892 (2)	171 (2)

Symmetry codes: (ii) −*x*+2, *y*+1/2, −*z*+1/2; (iii) *x*−1, *y*, *z*; (iv) −*x*+1, *y*+1/2, −*z*+1/2; (v) *x*, −*y*+1/2, *z*+1/2.

## References

[bb1] Bruker (1998). *SMART*, *SAINT* and *SADABS* Bruker AXS Inc., Madison, Wisconsin, USA.

[bb2] He, X., Lu, C.-Z. & Yuan, D.-Q. (2006). *Inorg. Chem.* **15**, 5760–5766. 10.1021/ic0520162 16841979

[bb3] Sheldrick, G. M. (2008). *Acta Cryst.* A**64**, 112–122. 10.1107/S0108767307043930 18156677

[bb4] Zhang, X.-M., Zhao, Y.-F., Zhang, W.-X. & Chen, X.-M. (2007). *Adv. Mater.* **19**, 2843–2846.

